# Susceptibilities of Nonhuman Primates to Chronic Wasting Disease

**DOI:** 10.3201/eid1509.090253

**Published:** 2009-09

**Authors:** Brent Race, Kimberly D. Meade-White, Michael W. Miller, Kent D. Barbian, Richard Rubenstein, Giuseppe LaFauci, Larisa Cervenakova, Cynthia Favara, Donald Gardner, Dan Long, Michael Parnell, James Striebel, Suzette A. Priola, Anne Ward, Elizabeth S. Williams, Richard Race, Bruce Chesebro

**Affiliations:** Rocky Mountain Laboratories, Hamilton, Montana, USA (B. Race, K.D. Meade-White, K.D. Barbian, C. Favara, D. Gardner, D. Long, M. Parnell, J. Striebel, S.A. Priola, A. Ward, R. Race, B. Chesebro); Colorado Division of Wildlife, Fort Collins, Colorado, USA (M.W. Miller); State University of New York Downstate Medical Center, Brooklyn, New York, USA (R. Rubenstein); New York State Institute for Basic Research in Developmental Disabilities, Staten Island, New York, USA (G. LaFauci); American Red Cross, Rockville, Maryland, USA (L. Cervenakova); University of Wyoming, Laramie, Wyoming, USA (E.S. Williams); 1 These authors contributed equally to this article.; 2 Deceased.; 3 Co-senior authors.

**Keywords:** Chronic wasting disease, oral transmission, intracerebral transmission, cynomolgus macaques, squirrel monkeys, TSE diseases, prions and related diseases, research

## Abstract

Chronic wasting disease (CWD) is a transmissible spongiform encephalopathy, or prion disease, that affects deer, elk, and moose. Human susceptibility to CWD remains unproven despite likely exposure to CWD-infected cervids. We used 2 nonhuman primate species, cynomolgus macaques and squirrel monkeys, as human models for CWD susceptibility. CWD was inoculated into these 2 species by intracerebral and oral routes. After intracerebral inoculation of squirrel monkeys, 7 of 8 CWD isolates induced a clinical wasting syndrome within 33–53 months. The monkeys’ brains showed spongiform encephalopathy and protease-resistant prion protein (PrPres) diagnostic of prion disease. After oral exposure, 2 squirrel monkeys had PrPres in brain, spleen, and lymph nodes at 69 months postinfection. In contrast, cynomolgus macaques have not shown evidence of clinical disease as of 70 months postinfection. Thus, these 2 species differed in susceptibility to CWD. Because humans are evolutionarily closer to macaques than to squirrel monkeys, they may also be resistant to CWD.

## Abstract 2

A species barrier may protect humans from this disease.

Transmissible spongiform encephalopathies (TSEs), or prion diseases, are neurodegenerative diseases that affect many mammalian species. Some examples include bovine spongiform encephalopathy (BSE) in cattle, scrapie in sheep and goats, Creutzfeldt-Jakob disease (CJD) in humans, and chronic wasting disease (CWD) in cervids. CWD was first found in captive deer in Colorado in 1967 (*1*) and was later identified in several US states and Canadian provinces (*2*). Epidemiologic evidence suggests that CWD continues to spread among cervid populations in North America (*3*), creating concern that CWD may cross species barriers to infect humans or domestic animals that may be eaten by humans. Thus, the host range of CWD and the level of protection provided by species barriers should be determined.

Substantial progress has been made in testing species barriers for CWD by using transgenic mice expressing species-specific prion protein (PrP), by direct infection into new species, or by in vitro conversion assays. The most sensitive method for testing susceptibility to TSE agents is intracerebral injection. Unfortunately, this route does not mimic most natural situations and only enables assessment of whether the possibility of transmission exists. Hamir et al. infected cattle and sheep with CWD by the intracerebral route and found protease-resistant PrP (PrPres) in 5 of 13 cattle and 2 of 8 sheep, which indicated that these ruminant species can propagate CWD (*4*,*5*). However, oral exposure in these hosts apparently does not cause disease (*2*).

CWD cross-species transmission to nonagricultural and laboratory animals has shown variable levels of susceptibility depending on the route of transmission. For example, ferrets were 100% susceptible to CWD by intracerebral infection but were not susceptible to oral infection (*6*,*7*). Mink were only 25% susceptible to CWD by intracerebral infection and were not susceptible to oral infection (*8*). CWD has been successfully transmitted and adapted to laboratory rodents, including hamsters, transgenic mice expressing hamster PrP, and transgenic mice overexpressing mouse PrP (*9*,*10*). In contrast, transgenic mice expressing human PrP were not susceptible to CWD by intracerebral infection (*11*,*12*), a finding that provided evidence for a human species barrier against CWD infection. However, work started in 1980 and published in 2005 by Marsh et al. showed that 2 squirrel monkeys (*Saimiri sciureus*) infected by the intracerebral route with brain homogenate from a single CWD-affected mule deer became clinically sick at 31 and 34 months postinfection, and both were positive for PrPres (*13*). This evidence that at least 1 species of nonhuman primate was susceptible to CWD weakened the conclusion that humans may be protected from CWD by a species barrier.

We addressed 4 questions raised by the original observation that squirrel monkeys are susceptible to CWD (*13*). First, we compared intracerebral and oral routes of infection. This comparison was of interest because the oral route is likely to be an important natural route of disease transmission, and susceptibility is known to be lower by this route in most models. Second, we compared 2 species of nonhuman primates, cynomolgus macaques (*Macaca fascicularis*) and squirrel monkeys, each of which has previously shown susceptibility to various human prion diseases (*14*–*16*). However, humans are believed to be evolutionarily closer to cynomolgus macaques than to squirrel monkeys (*17*), and cynomolgus macaques may be a more accurate model for a human species barrier. Third, because only 1 CWD source was tested by Marsh et al. (*13*), we studied 8 different pools of CWD representing wild and captive cervids, including mule deer, white-tailed deer, and elk, from separate regions in the United States. Fourth, we tested the species tropism of CWD agent passaged in squirrel monkeys.

## Materials and Methods

A description of the materials and methods used in this study follows. Additional details are available in the Technical Appendix

### Animal Research

All monkeys and mice were housed at the Rocky Mountain Laboratories (Hamilton, MT, USA). Experimentation followed protocols approved by the National Institutes of Health Rocky Mountain Laboratories Animal Care and Use Committee.

### CWD Pools for Infection of Primates

CWD-positive brain homogenates were provided by E.S.W. and M.W.M. Contents of each pool were as follows: MD-1, 6 free-ranging mule deer from Wyoming (*18*); MD-2, 4 captive mule deer from Colorado; MD-3, 28 captive mule deer from Wyoming and Colorado (*2*,*19*); WTD-1, 7 captive white-tailed deer from Wyoming and Colorado (*18**,**20*); WTD-2, 1 wild white-tailed deer from Wyoming; Elk-1, 2 free-ranging elk from Wyoming (*18*); Elk-2, 6 elk from a South Dakota game farm; and Elk-3, 10 captive elk from Wyoming and Colorado. Normal elk brain was a pool from 2 elk from Montana obtained from Lynn Creekmore of the US Department of Agriculture.

### Inoculation of Monkeys

For intracerebral injections, squirrel monkeys received either 2 mg or 20 mg brain in a total volume of 200 μL, and cynomolgus macaques received 5 mg in a total volume of 500 μL. Oral doses of 200 mg brain/mL were given on 5 different days at 2–6 day intervals. Squirrel monkeys received 3-mL doses; most macaques received 4-mL doses. The inoculum was given to anesthetized animals through a rubber gastric tube.

### Inoculation of Transgenic Mice

Brain homogenates diluted in phosphate buffered balanced solution containing 2% fetal bovine serum were inoculated intracerebrally into young adult mice. Volumes were 50 μL.

### Generation of Transgenic Mice Expressing Human PrP

Mice expressing human PrP (tgRM and tg66) were generated by using a transgene, cosSHa.HumPrP, which was created by ligating the human PrP open reading frame into the cosSHa.Tet vector (*21*). The transgene was inoculated into eggs of FVBn–mouse PrP null mice in the laboratories of R.R. (tg66) and L.C. (tgRM). Each line of mice overexpressed human PrP as tested by Western blot with monoclonal antibody 3F4.

### Analysis of Protease-sensitive PrP and PrPres by Immunoblot

Tissues were prepared by making a 20% (wt/vol) homogenate in 0.01 M Tris buffer, pH 7.4. Samples to be analyzed for protease-sensitive PrP (PrPsen) contained the following protease inhibitors: 10 μmol/L leupeptin, 1 μmol/L pepstatin A, and 1 μg/mL aprotinin. Samples were sonicated for 1 min and centrifuged at 5,000 rpm for 10 min. Supernatants were mixed 1:1 in 2× sample buffer and boiled for 3 min before electrophoresis.

Preparation of samples for PrPres analysis has been described (*18*). Removal of carbohydrate residues from PrPres was performed by digestion with peptide-N-glycosidase F (*22*).

After electrophoresis, proteins were transferred to Immobilon polyvinylidene difluoride–P membranes (Millipore, Billerica, MA, USA), and PrP bands were detected with antibodies 3F4 (residues 109–112) (*23*), D13 (residues 96–106) (*24*) (InPro Biotechnology, Inc., South San Francisco, CA, USA), or L42 (residues 145–163) (r-Biopharm, Darmstadt, Germany) (*25*). Bands were detected by using enhanced chemiluminescence substrate (GE Healthcare, Piscataway, NJ, USA).

### Histopathologic and Immunohistochemical Analyses

Routine formalin fixation, embedding, and tissue-sectioning protocols were followed. Tissues were stained with hematoxylin and eosin and analyzed for pathologic changes. Immunohistochemical staining was performed by using an automated Nexus stainer (Ventana, Tucson, AZ, USA). Anti-PrP antibodies D13 and 3F4 were used for PrPres immunostaining as described (*26*,*27*).

### Sequencing

Primate genomic DNA was purified from whole blood, and PCR products were amplified by using PuRe Taq Ready-To-Go PCR beads (GE Healthcare). Two primers from the extreme outer ends of the open reading frame, including the previously published forward primer HM-1 (*28*) with mPrP-780R (5′-TCCCACTATCAGGAAGATGAGG-3′) or a combination of outer primers with internal primers mPrP-397F (5′-CCTTGGTGGCTACATGCTG-3′) and mPrP-416R (5′-CCAGCATGTAGCCACCAAG-3′), were used. Assembly comparisons were made against human, elk, mule deer, cynomolgus macaque, and squirrel monkey by using Sequencher version 4.6 (Gene Codes, Ann Arbor, MI, USA).

## Results

### Infectivity Levels in CWD Pools

**Figure 1:**
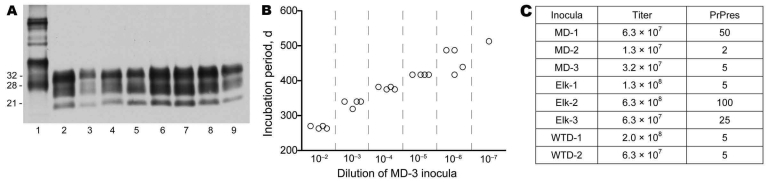
A) Western blot of chronic wasting disease (CWD) inocula showing protease-resistant prion protein (PrPres) in 8 CWD brain homogenate pools used for infecting nonhuman primates. Lane 1, 0.2-mg tissue equivalents of uninfected elk brain not treated with proteinase K; lanes 2–9, samples treated with proteinase K: lanes 2, 6, and 7, 0.12-mg tissue equivalents; lanes 3–5, 8, and 9, 0.67 mg tissue equivalents. PrPres was detected by using antibody L42 against PrP and enhanced chemiluminescence (GE Healthcare, Piscataway, NJ, USA). To provide optimal exposure for viewing PrP in all lanes, blot was exposed to film for 20 min. In this exposure, lanes 2, 6, 7, and 8 were exposed beyond the linear range; this blot could not be used to quantify relative PrPres levels. Values on the left are in kDa. For more accurate quantitations of PrPres, other gels with different amounts loaded were exposed for multiple times (see panel C). B) Titration of MD-3 CWD inoculum. End-point infectivity titrations were calculated for each CWD inoculum by inoculating 50 μL of serial 10-fold dilutions of each brain homogenate into transgenic mice expressing deer PrP, starting with a 1% (10^–2^) brain homogenate. Shown are data for an MD-3 inoculum. As the inoculum became more dilute, the incubation period (in days) and variability within a group increased. Each open circle represents 1 mouse in which clinical CWD developed. One mouse inoculated with a 10^–6^ dilution and 5 mice inoculated with a 10^–7^ dilution did not become sick after 625 days (solid circles). C) Infectivity titer and PrPres levels of each CWD pool. Titers are 50% infectious dose/g of brain homogenate. Relative level (%) of PrPres in each pool was measured by Western blot with a combination of serial dilutions and sequential exposure times in the linear response range for each sample. Data obtained from these comparisons are summarized in the PrPres column. All pools were compared with the pool with the highest PrPres signal (Elk-2), which was set at 100%.

When the 8 pools of CWD (representing both wild and captive deer and elk) used as inocula were analyzed by immunoblot, PrPres in the 8 pools showed similar electrophoretic mobilities and glycoform patterns (Figure 1, panel A), but PrPres levels differed when quantitatively compared (Figure 1, panel C). To measure the level of infectivity in these pools, we titered each pool in transgenic mice expressing deer PrP (line 33; tgDeerPrP) (*18*). A typical endpoint dilution titration is shown in Figure 1, panel B. The 8 pools had 50% infectious dose (ID_50_) titers ranging from 6.3 × 10^7^ to 5.0 × 10^8^ ID_50_/g of brain homogenate (Figure 1, panel C). Comparison of titers with PrPres levels showed a partial correlation (Figure 1, panel C). For example, the CWD pool with the lowest infectivity titer (MD-2) was also the pool with the lowest PrPres level. However, for some pools, these tests showed discrepant values.

### Intracerebral Infection of Squirrel Monkeys

To test susceptibility to CWD, we inoculated squirrel monkeys with each of the 8 CWD pools described above. Of 13 squirrel monkeys, 11 became symptomatic (33–53 mo postinfection [mpi]) (Table 1). The most consistent and reliable clinical finding was a severe wasting syndrome. Weight loss (average decrease of 33%) was most pronounced in the final few months of infection. Affected monkeys also had rough, poor-quality coats despite continuing to eat and drink. In the final 3–5 weeks, monkeys became weak and less active and spent most of their time hunched at the bottom of their cage. When the monkeys were encouraged to move, they did so slowly and deliberately. In the terminal stage of disease, a few had muscle tremors, excessive salivation, and mild ataxia. Fine, coordinated movement such as eating food was rarely affected. Monkeys were euthanized when terminal-stage weakness and wasting compromised their mobility and ability to eat and drink.

**Table 1:** Results of squirrel monkey intracerebral inoculation with CWD agent*

Monkey no.†	PrP genotype‡	CWD inoculum	Titer inoculated§	Incubation period, mpi¶	Weight change, %
308	NT	MD-1	1.0 × 10^6^	36	–8
633	A	MD-1	1.0 × 10^7^	36	–42
334	B	MD-2	6.4 × 10^5^	43	–38
393	B	MD-2	6.4 × 10^5^	46	–28
640	A	MD-3	2.0 × 10^6^	44	–35
365	NT	Elk-1	1.3 × 10^5^	40	–43
643	A	Elk-1	1.3 × 10^6^	53	–27
321	NT	Elk-2	4.0 × 10^5^	35	–23
322	NT	Elk-3	2.6 × 10^5^	33	–40
624	A	Elk-3	2.6 × 10^6^	48	–37
399	A	WTD-1	8.0 × 10^6^	50	–33
628	NT	WTD-1	8.0 × 10^6^	NS (52)	0
310	A	WTD-2	1.3 × 10^5^	NS (69)	+7
319	A	Normal elk		NS (69)	–8

*CWD, chronic wasting disease; PrP, prion protein; mpi, months postinfection; NT, not tested (sequenced); NS, no signs. †In addition to the monkeys listed, 4 asymptomatic squirrel monkeys were euthanized at 10 mo after intracerebral inoculation with MD-1, MD-3, Elk-1, and WTD-1 to detect early accumulation of protease-resistant PrP (PrPres), but no PrPres was detected in brain by Western blot. ‡See Table 4 for a description of genotypes A, B, and C. §Infectivity titers were determined by using endpoint dilution titer in transgenic deer expressing mouse PrP and are the 50% infectious dose/g of brain. ¶Incubation periods for monkeys with clinical wasting are indicated as mpi in parentheses. NS indicates that these monkeys did not show any clinical signs compatible with transmissible spongiform encephalopathy or wasting.

No clear correlation between incubation period and amount of agent inoculated was noted (Table 1). For example, 3 pairs of monkeys received the same inocula but in amounts that differed by 10-fold (Elk-1, Elk-3, and MD-1). Two pairs that received the lower dose became clinically sick first (Elk-1 and Elk-3). Both members of the third pair (MD-1) were euthanized after 36 months (Table 1). Two animals received the same dose of WTD-1 pool, yet to date, only 1 animal has become clinically sick. Animals that received the CWD pool with the lowest titer (MD-2) had incubation periods similar to those receiving much higher titered inocula (Table 1).

**Figure 2:**
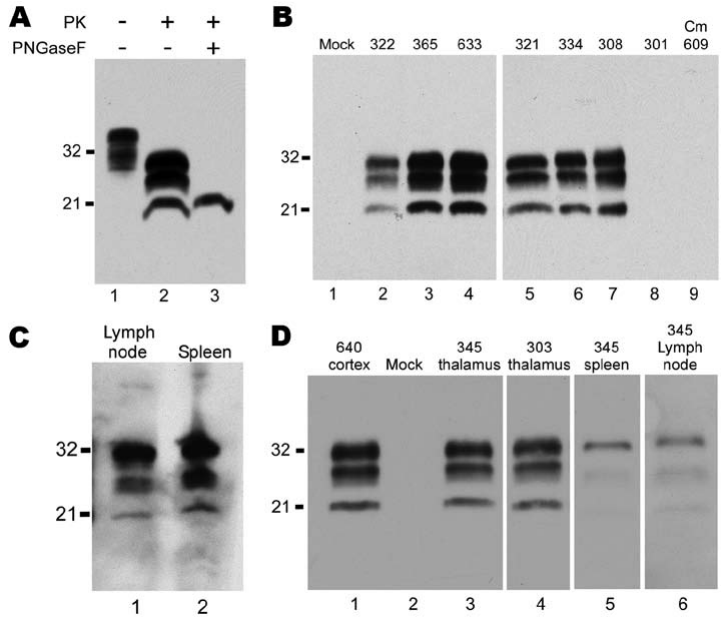
Western blots of squirrel monkey protease-resistant prion protein (PrPres). A) Brain homogenate from squirrel monkey 322, showing proteinase K (PK)–resistant PrPres. A downward shift of 7–9 kDa after PK digestion indicated a banding pattern typical of PrPres (lane 2). After deglycosylation with peptide-N-glycosidase F, 1 band of PrPres was present (lane 3). Lane 1, 0.5 mg of brain tissue equivalents; lane 2, 0.6 mg; lane 3, 0.4 mg. Blot was developed by using antibody 3F4 against PrP, enhanced chemiluminescence (ECL), and a 2-min exposure. B) Brains of monkeys screened for PrPres. All tissues were treated with PK, and lanes were loaded with 0.25 mg brain tissue equivalents, except for lane 5, which was loaded with 1.0 mg. All lanes contain samples of brain cortex except lanes 6 and 7, which contain thalamus. Blot was developed by using antibody 3F4, ECL, and a 30-min exposure. Lane 1, control. In lanes 2–7, PrP banding is similar among squirrel monkeys infected with different pools of chronic wasting disease (CWD) agent (Table 1). PrPres was not detected in brain of orally infected squirrel monkey 301 (lane 8) or in brain of an intracerebrally infected cynomolgus macaque (Cm) 609 (lane 9). C) Lymphatic tissues from squirrel monkey 365. For visualization of PrPres in lymph node and spleen, increased amounts of tissue were loaded, and a more sensitive detection system (femto detection; Thermo Scientific, Waltham, MA, USA) was used. Lane 1, lymph node, 0.7 mg tissue equivalents; lane 2, spleen, 1.1 mg tissue equivalents. Blot was developed by using antibody 3F4, femto-enhanced ECL, and a 1-min exposure. D) PK-treated brain and lymphatic tissues from orally infected squirrel monkeys 303 and 345. Lane 1, positive control no. 640; lane 2, negative control; lane 3, no. 345 thalamus; lane 4, no. 303 thalamus; lane 5, no. 345 spleen; lane 6, no. 345 mesenteric lymph node. Bands were visualized by using antibody 3F4 (residues 109–112) and ECL. Lanes 1–5, 10-min exposure; lane 6, overnight exposure; tissue equivalents loaded per lane: lanes 1–4, 0.25 mg; lane 5, 0.5 mg; lane 6, 1 mg. Values on the left of all blots are in kDa.

In all monkeys with clinical signs, CWD was confirmed by Western blot detection of PrPres in brain (Figure 2, panels A, B). The glycoform pattern of PrPres was similar for all affected monkeys inoculated with different CWD pools (Figure 2, panel B). Because PrPres deposition may also occur outside the central nervous system, we also tested peripheral lymphoid tissues. For 3 of 11 monkeys that had PrPres in brain, PrPres was also found in spleen and lymph nodes (Figure 2, panel C). In general, PrPres levels were much lower in lymphoid tissues than in brain and were often not detected by Western blot. All nonlymphatic tissues tested (cardiac muscle, skeletal muscle, duodenum, jejunum, ileum, colon, salivary gland, kidney, and lung) were negative for PrPres by immunoblot.

**Figure 3:**
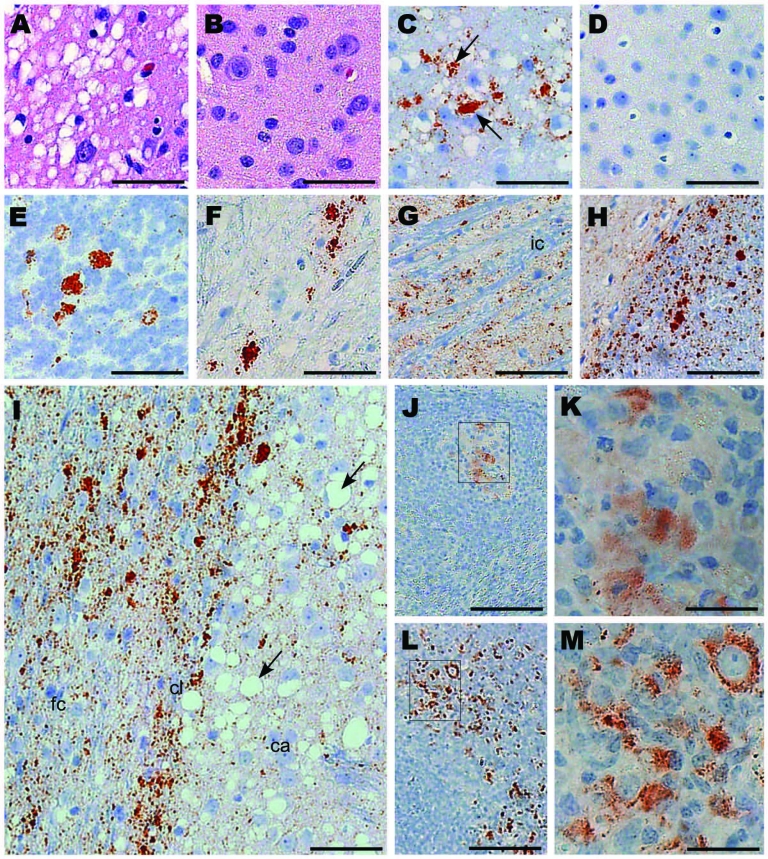
Immunohistochemical analysis of squirrel monkeys infected with chronic wasting disease (CWD) agent. Panels A, C, and E–M are from squirrel monkeys infected with CWD. Panels B and D are from an uninfected monkey showing no pathologic changes or positive staining for protease-resistant prion protein (PrPres). Panels A and B, cerebral cortex stained with hematoxylin and eosin; panels C and D, thalamus stained with antibody 3F4 against PrP (arrows); panels E and F, cerebellar granular cell layer and spinal cord, respectively, stained with antibody 3F4; panel G, gray matter within the internal capsule stained with antibody D13 against PrP; panel H, corpus callosum (right) stained with antibody D13 showing more intense staining than the adjacent cortex (left); panel I, frontal cortex (fc), claustrum (cl), and caudate (ca) stained with antibody 3F4 (abundant vacuoles in the putamen [arrows]); panels J–M, lymphatic tissue stained with antibody 3F4; panels J and K, PrPres staining in spleen of monkey 322; panels L and M, PrPres-positive mesenteric lymph node from orally infected monkey 301. Rectangles in panels J and L show areas enlarged in panels K and M, respectively. Antibodies D13 and 3F4 showed similar results for each monkey regarding the distribution, characteristics, and plaque size of PrPres. Scale bars: panels A–I, 50 μm; panel J, 100 μm; panels K and M, 25 μm; panel L, 250 μm.

Tissues from squirrel monkeys euthanized after intracerebral injection with CWD (Table 1) were also examined by histopathologic analysis, including staining with hematoxylin and eosin and immunohistochemical detection of PrPres. All monkeys examined had spongiosis in the cerebral cortex, caudate, putamen, and thalamus (Figure 3, panel A). In addition, PrPres deposition was observed in many brain regions with large PrPres-positive plaques in the thalamus, cerebellum, and spinal cord (Figure 3, panels C, E, F) and in smaller plaques spread out in the gray matter of the internal capsule and white matter of the corpus callosum (Figure 3, panels G, H). The most abundant and consistent location for PrPres staining was found in the frontal cortex and in the fiber tracts of the claustrum (Figure 3, panel I). The adjacent caudate had severe spongiosis and astrocytosis but minimal PrPres (Figure 3, panel I). PrPres was also detected in lymph nodes and spleen, within follicles, in areas resembling follicular dendritic cells (Figure 3, panels K, M). Immunohistochemical analysis showed no PrPres in heart, kidney, adrenal gland, skeletal muscle, salivary gland, tongue, pancreas, white fat, and all regions of the gastrointestinal tract.

### Oral Infection of Squirrel Monkeys

To test a more natural route of infection, we exposed squirrel monkeys orally to CWD. Of the 15 exposed squirrel monkeys, 1 (no. 345) was found dead in its cage at 69 mpi; it had shown no neurologic signs or weakness. Western blot results indicated PrPres in brain, spleen, and lymph nodes (Figure 2, panel D). The level of PrPres in the brain of monkey 345 was comparable with that in end-stage intracerebrally inoculated monkeys; body weight at necropsy indicated a 33% decrease over the final 10 months. The high levels of PrPres and the severe wasting indicate that CWD infection could have been the cause of death. A second monkey, 303, was euthanized at 69 mpi because of suspicion of TSE after 2 weeks of progressive weakness, wasting, and eventual anorexia. PrPres analysis confirmed PrPres in brain (Figure 2, panel D), spleen, and lymph nodes. For monkeys 303 and 345, levels of PrPres in the lymph nodes and spleens were 10–100-fold lower than those in brain.

Two other orally infected monkeys were euthanized during the first 69 mpi (Table 2). Monkey 301 was euthanized at 39 mpi, after rapid onset of lethargy and anorexia that led to severe dehydration. Results of Western blot analysis for PrPres were negative in brain (Figure 1, panel B), spleen, lymph nodes, heart, skeletal muscle, duodenum, jejunum, ileum, colon, salivary gland, kidney, lung, and tonsil. However, immunohistochemical analysis detected PrPres in the spleen and 1 mesenteric lymph node from this monkey, indicating a low level of infection (Figure 3, panels J,K). Monkey 614 was euthanized at 44 mpi because it did not recover from anesthesia related to routine tuberculosis screening. Neither Western blot nor immunohistochemical analysis detected PrPres in brain, spleen, or lymph nodes of this monkey.

**Table 2:** Results of squirrel monkey oral inoculation with CWD agent*

Monkey no.	PrP genotype†	CWD inoculum	Titer inoculated‡	Incubation period, mpi§	Weight change, %
303¶	NT	MD-1	1.5 × 10^9^	69	–25
360	A	MD-1	1.5 × 10^9^	NS (69)	+6
588	C	MD-3	9.6 × 10^7^	NS (52)	+5
629	B	MD-3	9.6 × 10^7^	NS (52)	0
631	A	Elk-1	1.9 × 10^8^	NS (52)	0
335	NT	Elk-2	6.0 × 10^8^	NS (69)	–5
656	B	Elk-2	6.0 × 10^8^	NS (52)	–5
614#	A	Elk-2	6.0 × 10^8^	44	–10
317	C	Elk-3	3.9 × 10^8^	NS (69)	0
301**	NT	Elk-3	3.9 × 10^8^	39	–14
307	A	WTD-1	1.2 × 10^9^	NS (69)	+8
345††	A	WTD-1	1.2 × 10^9^	69	–33
626	NT	WTD-2	1.9 × 10^8^	NS (52)	+11
641	B	WTD-2	1.9 × 10^8^	NS (52)	0
325	NT	WTD-2	1.9 × 10^8^	NS (69)	–8
655	A	Buffer control		NS (52)	–6
314	NT	Normal elk		NS (69)	+7

*CWD, chronic wasting disease; PrP, prion protein; mpi, months postinfection; NT, not tested (sequenced); NS, no signs. †See Table 4 for a description of genotypes A, B, and C. ‡Infectivity titers were determined by using endpoint dilution titer in transgenic deer expressing mouse PrP and are listed as the 50% infectious dose/g of brain administered over 5 d at 2–6-d intervals. §Incubation periods for monkeys with clinical wasting are indicated as mpi in parentheses. NS indicates that these monkeys did not show any clinical signs compatible with transmissible spongiform encephalopathy (TSE) or wasting. ¶Monkey 303 was euthanized at 69 mpi because of signs of wasting, weakness, and anorexia. #Monkey 614 was euthanized at 44 mpi because of complications arising from anesthesia for routine tuberculosis testing. No signs of TSE were observed before the complications, and Western blot and immunohistochemical results showed that this monkey was negative for protease-resistant PrP (PrPres). **Monkey 301 was euthanized at 39 mpi after a brief illness with signs of lethargy, anorexia, and dehydration. PrPres was detected in peripheral lymphoid tissues but not in brain. ††Monkey 345 was found dead at 69 mpi. Brain, spleen, and lymph nodes were positive for PrPres by Western blot.

### Infection of Cynomolgus Macaques

We inoculated cynomolgus macaques both orally and intracerebrally with 3 CWD inocula representing elk, mule deer, and white-tailed deer (Table 3). Of the cynomolgus macaques, 1 (no. 609) was euthanized at 48 mpi after it became aggressive. Brain (Figure 2, panel B), spinal cord, spleen, and lymph nodes were negative for PrPres by Western blot and immunohistochemical analysis. All remaining CWD-inoculated cynomolgus monkeys are currently (at 70 mpi) neurologically asymptomatic and have stable or increased body weights.

**Table 3:** Cynomolgus macaques infected with CWD agent*

No. monkeys	CWD inoculum	Route of inoculation	Titer inoculated†	Current mpi
1 (no. 609)‡	MD-1	Intracerebral	2.5 × 10^6^	NA
1	MD-1	Intracerebral	2.5 × 10^6^	70
3	MD-1	Oral	2.0 × 10^9^	70
2	Elk-1	Intracerebral	3.2 × 10^5^	70
3	Elk-1	Oral	2.5 × 10^8^	70
2	WTD-1	Intracerebral	1.0 × 10^6^	70
3	WTD-1	Oral	1.2 × 10^9^	70

*CWD, chronic wasting disease; mpi, months postinfection; NA, not available. †Infectivity titers were determined by using endpoint dilution titer in transgenic deer expressing mouse prion protein and are listed as the total 50% infectious dose/g of brain. ‡Monkey 609 was euthanized at 48 mpi, and no protease-resistant prion protein was detected by Western blot or immunohistochemical analysis.

### Sequences

Amino acid substitutions in PrP can alter susceptibility to TSE agents, including CWD (*18*,*29*,*30*). To determine whether the lack of susceptibility in several intracerebrally inoculated squirrel monkeys (Table 1) was caused by PrP gene polymorphisms, we sequenced the PrP genes from 23 squirrel monkeys. When compared with published squirrel monkey sequences (*28*,*31*), variation was seen at residue 164, in the number of octapeptide repeats, and at residue 19 of the signal peptide (Table 4). However, these genetic differences in PrP did not appear to account for the lack of susceptibility of monkey 310, which was genotype A, because this genotype was also found in 5 of the CWD-positive monkeys. Because we were not able to sequence PrP of monkey 628, we could not assess the role of PrP variation in the lack of disease.

**Table 4:** PrP sequence variability in squirrel monkeys*

Genotype†	No. monkeys	PrP gene variations‡		
		Residue 19	No. octapeptide repeats	Residue 164
A	16	Leu/Leu	5/5	Lys/Lys
B	5	ND	4/5	Lys/Lys
C	2	Val/Leu	5/5	Lys/Lys
Schätzl	1	Leu/Leu	5/5	Arg/Arg
Schneider	1	ND	4/4	Arg/Arg

*PrP, prion protein; ND, not determined. †The PrP genes of 23 monkeys were sequenced, and 3 genotypes were found. For easy reference to Tables 1 and 2, they are designated types A, B, and C. Previous squirrel monkey PrP sequences were identified by Schätzl et al. (*28*) and Schneider et al. (*31*). ‡Compared with published sequences, PrP genotype variations were seen only at residue 19 (in the signal peptide), residue 164, and in the number of octapeptide repeats.

### Infectivity of CWD-infected Squirrel Monkey Tissues in PrP Transgenic Mice

To determine whether passage of CWD in squirrel monkeys altered the tropism of the infectious agent, we inoculated tgDeerPrP mice and tg mice expressing human PrP (lines 66 and RM) intracerebrally with tissue homogenates from 3 CWD-positive squirrel monkeys (nos. 322, 308, and 301) with PrPres and from an intracerebrally inoculated cynomolgus macaque (no. 609). Clinical disease did not develop in any tgDeerPrP, tg66, or tgRM mice during 600–700 days (Table 5). The lack of transmission to tgDeerPrP mice from the 3 squirrel monkeys with detectable CWD PrPres indicated that either the infectivity levels were low in these squirrel monkeys or that the original cervid species tropism was altered by the passage in squirrel monkeys. Similarly, the lack of transmission to tg mice expressing human PrP implied that passage through squirrel monkeys did not facilitate adaptation to an agent with increased tropism for humans.

**Table 5:** Infectivity of CWD agent from cervids, squirrel monkeys, and cynomolgus macaques in transgenic mice expressing deer PrP or human PrP*

Donor†	Original inoculum	Donor PrPres‡	TSE disease incidence§		
			tg33 (deer)	tg66 (human)	tgRM (human)
SM 322¶	Elk-3	+	0/8	NT	0/6
SM 308¶	MD-1	+	0/7	0/8	0/8
SM 301	Elk-3	±	0/6	NT	NT
SM 320	Uninfected	–	0/7	NT	NT
CM 609	MD-1	–	0/8	NT	NT
Elk-3	NA	+	6/6 (301 ± 11)	NT	NT
MD-1	NA	+	7/7 (323 ± 15)	NT	NT
sCJD (97–008)	NA	+	NT	NT	4/6 (170 ± 3)
sCJD (99–009)	NA	+	NT	NT	5/5 (194 ± 20)
sCJD (RR)	NA	+	NT	8/8 (163 ± 1)	NT
sCJD (PLG)	NA	+	NT	4/4 (163 ± 6)	NT

*CWD, chronic wasting disease; PrP, prion protein; TSE, transmissible spongiform encephalopathy; NT, not tested; sCJD, sporadic Creutzfeldt-Jakob disease. †Each donor monkey inoculum was prepared as a 1% brain homogenate from the indicated monkeys. SM, squirrel monkey; CM, cynomolgus macaque. Elk and mule deer CWD inocula were described in Materials and Methods. Human sCJD inocula are brain homogenates from World Health Organization CJD reference materials. No. 99–009 is sCJD M/M type I, and no. 97–008 is sCJD M/M type II. The RR sample was from a patient with sCJD of unknown PrP genotype. The PLG sample (M/M type I) was from a patient with sCJD. In all cases except sCJD (RR), 50 μL was inoculated intracerebrally into recipient mice; for sCJD (RR), 30 μL was inoculated. ‡Based on Western blot or immunohistochemical analysis of brain for all except monkey 301, in which protease-resistant PrP (PrPres) was detected in spleen but not brain. §Number of recipient mice with clinical transmissible spongiform encephalopathy confirmed by detection of brain PrPres is the numerator, and total number of mice inoculated is the denominator. Mean ± SEM incubation period for time to clinical disease is provided in days. Tg33 mice express deer PrP, and tg66 and tgRM mice express human PrP. ¶In addition to brain homogenate, we also inoculated tg33 mice with homogenates of spleen, lymph nodes, heart, muscle, and plasma from squirrel monkeys 322, 308, and 321, but disease did not develop during >600 d observation.

## Discussion

As new CWD foci continue to emerge among cervid populations, the risk for CWD transmission to humans needs to be assessed. We used 2 monkey species and 2 routes of inoculation to test the susceptibility of primates to 8 different pools of CWD. To date, we have verified CWD in 11 of 13 intracerebrally inoculated squirrel monkeys; average incubation period was 41 months (range 33–53 months). Using a single CWD pool, Marsh et al. noted infection in 2 of 2 squirrel monkeys 31–34 months after intracerebral inoculation (*13*). Intracerebral inoculation of squirrel monkeys with other TSE agents, including agents of kuru, variant CJD, sporadic CJD, and sheep scrapie, had incubation periods of ≈24 months and attack rates of ≈100% (*14*,*15*,*32*). The extended incubation periods and lower attack rates for our squirrel monkeys may result from a partial species barrier to CWD.

The signs of wasting syndrome in CWD-infected monkeys were similar to those of CWD infection in cervids, in which loss of body condition is nearly always a major component of infection and neurologic deficits vary (*2*). The correlation of clinical signs between CWD in cervids and squirrel monkeys suggests that CWD might affect a common brain region in each species. We observed PrPres deposition in squirrel monkeys primarily in the frontal lobe of the cerebral cortex, claustrum, putamen, and thalamus. Cervids typically have the most abundant and predictable PrPres in the dorsal motor vagus nucleus (obex), olfactory cortex, and diencephalon (including thalamus, hypothalamus, metathalamus, and epithalamus) (*2*,*33*). A plausible hypothesis could be that disruption of regions within the hypothalamus and thalamus leads to a metabolic imbalance, resulting in a severe wasting syndrome.

We did not observe a strong correlation between infectivity titer inoculated and attack incidence or incubation period (Table 1). One potential explanation is that the variation in speed of disease progression might not be relevant given the low number of animals in each group. A second possibility is that our squirrel monkeys varied at PrP alleles that may affect CWD susceptibility. However, analysis of 23 squirrel monkeys showed no PrP sequence differences correlating with susceptibility to CWD (Tables 1, 2, 4). A third possibility is that genes other than the gene for PrP might influence CWD susceptibility.

For humans, eating infected or contaminated tissue is a likely route of CWD exposure. In other animal models, oral transmission of TSE is generally 1,000-fold less effective than direct intracerebral challenge and results in longer incubation periods and lower efficiency of disease transmission. In our oral transmission experiments, we found evidence of CWD infection in 3 monkeys; 2 at 69 mpi had abundant PrPres in brain and lower levels in spleen and lymph nodes, and 1 euthanized at 39 mpi had PrPres in lymphatic tissues only. Thus, transmission seems to be slower by the oral route than by the intracerebral route, and other orally infected monkeys may be affected in the future.

Cynomolgus macaques are evolutionarily closer to humans than are squirrel monkeys (*17*). At nearly 6 years postinoculation, no macaques have shown clinical signs of CWD. Intracerebral inoculation of cynomolgus macaques with BSE causes disease in 3 years; human variant CJD requires 2–3 years, and human sporadic CJD requires 5 years (*16*,*34*). However, oral inoculation of cynomolgus macaques with BSE agent required a minimum of 5 years before clinical disease was observed (*35*). Therefore, we cannot rule out CWD transmission to cynomolgus macaques.

**Figure 4:**
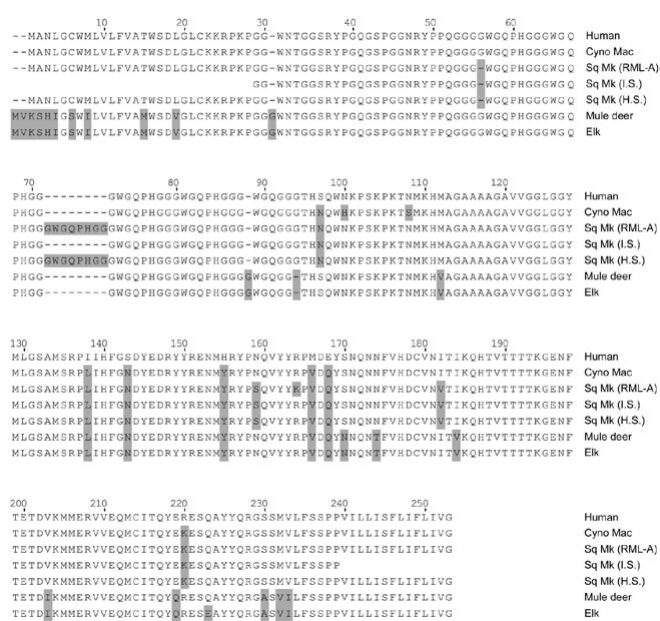
Comparison of prion protein sequences from various species. The following species are shown, and GenBank accession numbers are given when available: human (M13899), cynomolgus macaque (Cyno Mac) (U08298), squirrel monkey (Sq Mk) (genotype RML-A, see Table 4), squirrel monkey from Schneider et al. (*31*) (AY765385), squirrel monkey from Schätzl et al. (*28*) (U08310), mule deer (AY330343), and elk (AF156183). Numbering is based on the human sequence. Gray boxes indicate residues different from human residues. Alignment of the sequences was conducted with MegAlign software (DNAstar/Lasergene, Madison, WI, USA).

The PrP gene sequence can influence cross-species transmission of prion disease. Therefore, we compared squirrel monkey and cynomolgus macaque PrP gene sequences to look for differences that might account for different susceptibilities of these monkeys to CWD. In the PrP gene excluding the signal peptide, deer differed from squirrel monkeys at 17 residues and from cynomolgus macaques at 16 residues, but 14 of these differing residues were identical in squirrel monkeys and macaques (Figure 4). Therefore, there are only 2 residues in cynomolgus macaques (100 and 108) and 3 residues in squirrel monkeys (56, 159 and 182) at which these monkeys differ from deer and also from each other. These residues might play a role in susceptibility differences seen in our study.

Human exposure to CWD-infected cervids in past decades is likely. The highest levels of prion infectivity are present in the central nervous system and lymphatic tissues of CWD-infected cervids; contamination of knives, saws, and muscles with these tissues can easy occur when processing game. Despite the likelihood of exposures, epidemiologic studies of humans living in CWD-endemic areas of Colorado and Wyoming during 1979–2001 have not shown any increases in human TSE cases (*36*,*37*). Ongoing studies by the Colorado Department of Public Health and Environmental Human Prion Disease Surveillance Program, in conjunction with the University of Colorado School of Medicine, have also concluded that no convincing cases of CWD transmission to humans have been detected in Colorado (*38*). However, if CWD in humans appears like a wasting syndrome similar to that observed in the squirrel monkeys in our study, affected persons might receive a diagnosis of a metabolic disorder and never be tested for TSE. Fortunately, additional laboratory data are consistent with the epidemiologic data, and these results support the conclusion that a species barrier protects humans from CWD infection (*11*–*13*,*20*,*36*,*37*).

## Supplementary Material

Technical Appendix Susceptibilities of Nonhuman Primates to Chronic Wasting Disease
