# Supplementary material for: Susceptibilities of Nonhuman Primates to Chronic Wasting Disease
Source: Emerg Infect Dis. 2009 Sep;15(9):1366–76. doi: 10.3201/eid1509.090253 (PMC2819871; doi:10.3201/eid1509.090253)
Supplement: Technical Appendix — Susceptibilities of Nonhuman Primates to Chronic Wasting Disease [file 09-0253_Techapp-s1.pdf]

Susceptibilities of Nonhuman Primates to Chronic Wasting Disease

Technical Appendix

Materials and Methods

Nonhuman Primates

All monkeys were housed individually at the Rocky Mountain Laboratories (RML) (Hamilton, MT, USA) in a facility accredited by the Association for Assessment and Accreditation of Laboratory Animal Care International (Frederick, MD, USA). Experimentation followed protocols approved by the National Institutes of Health RML Animal Care and Use Committee. All monkeys were included in a nonhuman primate enrichment program that included housing in which they could view others in the colony and toys and treats for psychological well-being. Initially, 12 cynomolgus macaques (age range 3–9 years) were obtained from the RML breeding colony, and 3 were obtained from the Mauritius Islands off the coast of Africa through Primate Products, Inc. (Miami, FL, USA). Imported animals were screened for tuberculosis, B virus (cercopithecine herpesvirus 1), herpes simplex virus 1, measles, simian retrovirus, and simian immunodeficiency virus. Cynomolgus macaques were fed Hi-Protein Monkey Diet (Nestle Purina Petcare Co., St. Louis, MO, USA) augmented with fresh fruit and monkey treats (Bio-Serv, Frenchtown, NJ, USA). All squirrel monkeys were obtained from Guyana, South America, through Worldwide Primates (Miami, FL, USA). These animals were screened for tuberculosis, saimirine herpesviruses, measles, and cytomegalovirus. Squirrel monkeys were fed a combination primate diet (Nestle Purina Petcare Co. and ZuPreem, Shawnee, KS, USA) augmented with fresh fruit. Water was provided ad libitum to all monkeys in the study.

CWD Pools for Infection of Primates

MD-1 is a pool from 6 free-ranging mule deer from Wyoming and was published as deer-2 (*1*). MD-2 is a pool from 4 captive mule deer from Colorado identified by the Colorado Department of Wildlife as 03 407541, 03 465081, 03 464506, and 03 460516. MD-3 is a pool

from 28 captive mule deer from Wyoming and Colorado (*2*,*3*), also referred to as deer-3 (*1*) and md-PrP^{CWD} (*4*).

WTD-1 is a pool from 7 captive white-tailed deer from Colorado and Wyoming, published as deer-1 (*1*) and wtd-PrP^{CWD} (*4*). WTD-2 is from 1 wild white-tailed deer from Wyoming. Elk-1 is a pool from 2 free-ranging elk from Wyoming published as elk (*1*). Elk-2 is a pool from 6 elk (nos. 98W615, 98W618, 98W2542, 98W9292, 98W9527, and 99W2206) from a South Dakota game farm. Elk-3 is a pool from 10 captive elk obtained from Colorado and Wyoming.

Normal elk brain was a pool from 2 animals from Montana, MTKS no. 37 and MTKS no. 49 (MTKS37/49), obtained from Lynn Creekmore of the U.S. Department of Agriculture. CWD pools MD-1, MD-3, WTD-1, WTD-2, Elk-1, Elk-2, and Elk-3 were contributed in full or in part by E.S.W., and pools MD-2, MD-3, WTD-1 and Elk-3 were contributed in full or in part by M.W.M.

Inocula were prepared by homogenizing brain tissue in 0.01 M Tris-HCl, pH 7.4, by using a sterile glass dounce with 10 strokes or until no clumps were visible. Pooled samples were combined in a 100-mL graduated cylinder on ice and adjusted to 20% wt/vol. Homogenates were vortexed and then sonicated for 2 min. For intracerebral infection, samples were cleared by centrifuging at 2,000 rpm for 5 min. Supernatants were frozen at −80°C until needed. Before infection, samples were thawed in a 37°C water bath, sonicated for 1 min, and diluted in phosphate-buffered balanced salt solution containing 2% fetal bovine serum to achieve concentrations needed for intracerebral inoculations.

Inoculation of Monkeys and Mice

For intracerebral inoculations, squirrel monkeys received either 2 mg or 20 mg brain in a total volume of 200 μL, and cynomolgus macaques received 5 mg in a total of 500 μL volume. The diluted brain homogenate was injected into a surgically created hole in the skull over the left parietal lobe of anesthetized animals.

Oral doses of 200 mg brain/mL were given on 5 different days at 2–6 day intervals. Squirrel monkeys received 3-mL doses; most macaques received 4-mL doses. Three macaques inoculated with WTD-1 received 3-mL doses. The inoculum was given to anesthetized animals

through a rubber gastric tube that was flushed with Tris-buffered saline (TBS) before removal from the stomach.

Intracerebral inoculations into the parietal lobe of anesthetized young adult mice were conducted by using tissue homogenates diluted in phosphate-buffered balanced salt solution containing 2% fetal bovine serum. Volumes were 50 µL unless otherwise indicated.

Titration and Passage of CWD Infectivity in Transgenic Mice

The 8 cervid pools were titered by endpoint dilution with serial 1:10 dilutions of brain homogenate ranging from 10^{-2} (1%) to 10^{-8}. Dilutions were inoculated intracerebrally into transgenic mice expressing deer PrP (line 33) (*1*). For all other inoculations into transgenic mice expressing deer or human PrP, a 10^{-2} dilution was used. The volume inoculated was 50 µL for all homogenates except for the sporadic CJD inoculum used in the tg66 mice, which was 30 µL. Mice were observed for clinical signs for >600 days (Figure 1, panel B). Mice with clinical signs were euthanized, and a diagnosis of CWD was confirmed by immunoblot detection of PrPres. End-point titer was determined by using the Spearman-Karber method (*5*). Briefly, the following formula was used: 50% infectious dose (ID_{50}) = $x_{p=1} + 1/2d - d\sum p$ where $x_{p=1}$ is the highest log dilution giving all positive responses, d is the log dilution factor, p is the proportion positive at a given dose, and Σp is the sum of values of p for $x_{p=1}$ and all higher dilutions. When 10-fold dilutions were used, the error with this method was±0.5 logs. The log value for the ID_{50} was converted to scientific notation for all figures and tables in this report.

Generation of Transgenic Mice Expressing Human PrP

Mice expressing human PrP were generated by using a transgene, cosSHa.HumPrP, which was created by ligating the human PrP open reading frame (ORF) into the cosSHa.Tet vector (*6*). Full-length human PrP ORF from human DNA was generated as an 803-bp PCR product by using the forward and reverse primers 5′-TGAGCGGCCGCGCAGTCATTATGGCGAACCTTG-3′ and 5′-TACTGAGTCGACCCTTCCTCATCCCACTATCAGG-3′. The PrP ORF was cloned into pGEM-5zR+ after digestion with *Not*I and *Sal*I. Sequence analysis confirmed the human PrP sequence. Addition of a Kozak translation initiation site and cloning into the cosSHa.Tet vector was performed by using techniques similar to those described (*7*). The transgene was inoculated into eggs of FVBn– mouse PrP null mice in the laboratories of R.R. and L.C. We used

homozygous tg66 mice and hemizygous tgRM mice. These mice overexpress human PrP as tested by Western blot with monoclonal antibody 3F4. When compared with Tg7 hamster PrP–expressing mice, tg66 mice had 5–10× overexpression and tgRM mice had 1–2× overexpression. We did not have any human brain tissue for direct comparison with human PrP expression, but antibody 3F4 is known to react with human and hamster PrP.

Analysis of PrPres by Immunoblot

Tissues were prepared by making a 20% (wt/vol) homogenate in ice cold 0.01 M Tris buffer, pH 7.4, by using either an Omni Tissue homogenizer with disposable hard tissue probe (Omni International, Marietta, GA, USA) or a pestle in a DNase/RNase–free 1.5-mL tube (Kontes Glass Co., Schenectady, NY, USA). Samples were sonicated for 1 min and kept frozen until analyzed. Samples to be analyzed for protease-sensitive PrP were prepared as above with addition of the following protease inhibitors: 10 μmol/L leupeptin, 1 μmol/L pepstatin A, and 1 μg/mL aprotinin. After sonication, samples were centrifuged at 5,000 rpm for 10 min, and supernatants were mixed 1:1 in 2× sample buffer and boiled for 3 min. Preparation of samples for PrPres analysis has been described (*18*). Briefly, 20 μL of a 20% (wt/vol) tissue homogenate without protease inhibitors was adjusted to a concentration of 100 mmol/L Tris-HCl, pH 8.3, 1% Triton X-100, and 1% sodium deoxycholate in a total volume of 31 μL. Samples were treated with 50 μg/mL of proteinase K for 45 min at 37°C. The reaction was stopped by adding 2 μL of 0.1 M phenylmethylsulfonyl fluoride, and samples were placed on ice for 5 min. An equal volume of 2× Laemmeli sample buffer (Bio-Rad, Hercules, CA, USA) was added, and samples were boiled for 5 min.

Removal of carbohydrate residues from PrPres was performed by digestion with PNGaseF (*8*). Briefly, 20 μL of PrPres samples in 2× Laemmeli sample buffer were boiled for 10 min, and 2 μL of 10× G7 buffer and NP40 and 2 μL of PNGase F were added. Samples were incubated overnight at 37°C and kept frozen at −20°C until analyzed by immunoblotting.

Samples were subjected to electrophoresis on a 16% sodium dodecyl sulfate–polyacrylamide gel, and proteins were transferred to Immobilon polyvinylidene difluoride–P membranes (Millipore, Billerica, MA, USA). PrP bands were detected with antibodies 3F4 (residues 109–112) (*9*), D13 (residues 96–106) (*10*) (InPro Biotechnology, Inc., South San Francisco, CA, USA), or L42 (residues 145–163) (r-Biopharm, Darmstadt, Germany) (*11*).

Membranes were incubated in primary antibody (diluted 1:5,000) in TBS-T for 1.5 hours, rinsed with TBS–Tween (TBS-T) buffer, and incubated with their appropriate horseradish peroxidase–conjugated secondary antibody (sheep anti-mouse immunoglobulin [Ig] G for 3F4 and L42, sheep antihuman IgG for D13) at a 1:5,000 dilution in TBS-T for 30 min. Bands were detected by using enhanced chemiluminescence substrate as recommended by the manufacturer (GE Healthcare, Piscataway, NJ, USA).

Histopathologic and Immunohistochemical Analyses

Tissues were placed in 3.7% phosphate-buffered formalin for 3–5 days before dehydration and embedding in paraffin. Serial 4-μm sections were cut by using a standard microtome (Leica Microsystems Inc., Bannockburn, IL, USA), placed on a positively charged glass slide, and dried overnight at 56°C. Sections were stained with hematoxylin and eosin and analyzed for pathologic changes. Immunohistochemical staining was performed by using an automated Nexus stainer (Ventana, Tucson, AZ, USA). Slides were deparaffinized and rehydrated in Tris-HCl, pH 7.5. Staining for antibodies against PrP was conducted by using antibodies D13 and 3F4 against PrP as previously described (*12*,*13*).

Sequencing

Primate genomic DNA was purified from whole blood by using the QIAamp DNA Blood Maxi Kit (QIAGEN, Valencia, CA, USA) as directed. PCR products were amplified by using PuRe Taq Ready-To-go PCR beads (GE Healthcare). Two primers from the extreme outer ends of the ORF, including the previously published forward primer HM-1 (*14*) with mPrP-780R (5′-TCCCACTATCAGGAAGATGAGG-3′) or a combination of outer primers with internal primers mPrP-397F (5′-CCTTGGTGGCTACATGCTG-3′) and mPrP-416R (5′-CCAGCATGTAGCCACCAAG-3′), were used. Three successful amplicons were generated and purified by using the QIAquick PCR (QIAGEN) as recommended and sequenced by using their respective forward and reverse PCR primers on an ABI 3730xl instrument (Applied Biosystems, Inc., Foster City, CA, USA). Sequence data were stored in the FINCH data management system (Geospiza, Seattle WA, USA). Assembly comparisons were made against human, elk, mule deer, cynomolgus macaque, and squirrel monkey by using Sequencher version 4.6 (Gene Codes, Ann Arbor, MI, USA).

References

1. Meade-White K, Race B, Trifilo M, Bossers A, Favara C, Lacasse R, et al. Resistance to chronic wasting disease in transgenic mice expressing a naturally occurring allelic variant of deer prion protein. J Virol. 2007;81:4533–9. PubMed DOI: 10.1128/JVI.02762-06

2. Williams ES. Chronic wasting disease. Vet Pathol. 2005;42:530–49. PubMed DOI: 10.1354/vp.42-5-530

3. Sigurdson CJ, Williams ES, Miller MW, Spraker TR, O'Rourke KI, Hoover EA. Oral transmission and early lymphoid trophism of chronic wasting disease PrPres in mule deer fawns (Odocoileus hemionus). J Gen Virol. 1999;80:2757–64. PubMed

4. Raymond GJ, Bossers A, Raymond LD, O'Rourke KI, McHolland LE, Bryant PK III, et al. Evidence of a molecular barrier limiting susceptibility of humans, cattle and sheep to chronic wasting disease. EMBO J. 2000;19:4425–30. PubMed DOI: 10.1093/emboj/19.17.4425

5. Dougherty RM. Animal virus titration techniques. In: Harris RJ, editor. Techniques in experimental virology. New York: Academic Press Inc; 1964. p. 183–6.

6. Scott MR, Kohler R, Foster D, Prusiner SB. Chimeric prion protein expression in cultured cells and transgenic mice. Protein Sci. 1992;1:986–97. PubMed DOI: 10.1002/pro.5560010804

7. Lafauci G, Carp RI, Meeker HC, Ye X, Kim JI, Natelli M, et al. Passage of chronic wasting disease prion into transgenic mice expressing Rocky Mountain elk (Cervus elaphus nelsoni) PrPC. J Gen Virol. 2006;87:3773–80. PubMed DOI: 10.1099/vir.0.82137-0

8. Race BL, Meade-White KD, Ward A, Jewell J, Miller MW, Williams ES, et al. Levels of abnormal prion protein in deer and elk with chronic wasting disease. Emerg Infect Dis. 2007;13:824–30. PubMed

9. Kascsak RJ, Rubenstein R, Merz PA, Tonna-DeMasi M, Fersko R, Carp RI, et al. Mouse monoclonal and polyclonal antibody to scrapie-associated fibril proteins. J Virol. 1987;61:3688–93. PubMed

10. Matsunaga Y, Peretz D, Williamson A, Burton D, Mehlhorn I, Groth D, et al. Cryptic epitopes in N-terminally truncated prion protein are exposed in the full-length molecule: dependence of conformation on pH. Proteins. 2001;44:110–8. PubMed DOI: 10.1002/prot.1077

11. Vorberg I, Buschmann A, Harmeyer S, Saalmuller A, Pfaff E, Groschup MH. A novel epitope for the specific detection of exogenous prion proteins in transgenic mice and transfected murine cell lines. Virology. 1999;255:26–31. PubMed DOI: 10.1006/viro.1998.9561

12. Race B, Meade-White K, Oldstone MB, Race R, Chesebro B. Detection of prion infectivity in fat tissues of scrapie-infected mice. PLoS Pathog. 2008;4:e1000232.

13. Kercher L, Favara C, Striebel JF, Lacasse R, Chesebro BJ. Prion protein expression differences in microglia and astroglia influence scrapie-induced neurodegeneration in the retina and brain of transgenic mice. J Virol. 2007;81:10340–51. PubMed DOI: 10.1128/JVI.00865-07

14. Schätzl HM, Da Costa M, Taylor L, Cohen FE, Prusiner SB. Prion protein variation among primates. J Mol Biol. 1995;245:362–74. PubMed DOI: 10.1006/jmbi.1994.0030
